# Advances in Autoimmune Epilepsy Associated with Antibodies, Their Potential Pathogenic Molecular Mechanisms, and Current Recommended Immunotherapies

**DOI:** 10.3389/fimmu.2017.00395

**Published:** 2017-04-25

**Authors:** Zhiwei Fang, Yunqi Yang, Xuan Chen, Weiwang Zhang, Yangmei Xie, Yinghui Chen, Zhenguo Liu, Weien Yuan

**Affiliations:** ^1^Department of Neurology, Xinhua Hospital Affiliated to the Medical School of Shanghai Jiao Tong University, Shanghai, China; ^2^School of Pharmacy, Shanghai Jiao Tong University, Shanghai, China; ^3^Zhiyuan College, Shanghai Jiao Tong University, Shanghai, China; ^4^Department of Neurology, Jinshan Hospital, Fudan University, Shanghai, China

**Keywords:** autoimmune epilepsy, pathogenic mechanism, guidelines, autoantibodies, immunotherapy

## Abstract

In this comprehensive article, we present an overview of some most common autoimmune antibodies believed to be potentially pathogenic for autoimmune epilepsies and elaborate their pathogenic mode of action in molecular levels based on the existing knowledge. Findings of the studies of immunemodulatory treatments for epilepsy are also discussed, and guidelines for immunotherapy are sorted out. We aim to summarize the emerging understanding of different pathogenic mechanisms of autoantibodies and clinical immunotherapy regimens to open up therapeutic possibilities for future optimum therapy. We conclude that early diagnosis of autoimmune epilepsy is of great significance, as early immune treatments have useful disease-modifying effects on some epilepsies and can facilitate the recovery.

## Introduction

Epilepsy is recognized globally as one of the most common neurological diseases, characterized by seizures and cognitive impairment (1, 2). With unknown causes, lots of epilepsy patients have a poor response to antiepileptic drugs (AEDs) and thus have to live a life with low quality (3). Accumulating evidences support the role of autoimmune-mediated factors in patients with AED-resistant seizures, which eventually help to crystallize the concept of autoimmune epilepsy (AE) (4). Nowadays, the spectrum of AE has been greatly expanded by the accumulating discoveries of new autoantibodies targeting against self-antigen. Not only do AE refer to some diseases in which epilepsy is the primary syndrome, like focal epilepsy, status epilepticus, and generalized epilepsy (5), they also represent some related autoimmune disorders (6, 7) and tumors (8, 9), where epilepsy is a secondary manifestation. As is reported in studies on epilepsy patients, individuals who are autoantibody-positive tend to show a worse response to AEDs than negative ones, implying an immune pathogenesis (10, 11). The identification of an immune basis in AE is very important because early diagnosis and immunotherapy may actually limit the duration or severity of the illness and could improve the outcome of epilepsy recovery (12–15).

Although the precise pathogenesis of AE is not fully understood, it is widely believed that antibodies targeting against intracellular antigens or neuronal surface antigens are the potential causes (16). Studies on the pathophysiology of epilepsy demonstrate that autoantibodies targeting against different antigens within the neuronal cell or on the plasma membrane, exert different pathophysiological effects on the central nervous system (CNS).

## AE Studies with Antibodies Specific for Plasma Membrane

### AE Associated with Voltage-Gated Potassium Channel (VGKC) Complex Antibodies

Widely expressed throughout the entire CNS, VGKCs are transmembrane channels specific for potassium and sensitive to voltage changes. They play a pivotal role in the resting membrane potentials and neuronal action potentials, thus are significant for the regulation of neuronal excitability, axonal conduction, and neurotransmitter release (17, 18).

Traditionally, it was thought that associated autoantibodies were directed against the VGKC (19, 20). However, further study made it clear that what these antibodies actually bind to was associated VGKC complex proteins, rather than VGKCs themselves (21). These associated proteins serve as antigenic targets in the diseases, including leucine-rich glioma inactivated 1 (LGI1), contactin-associated protein 2 (CASPR2), contactin-2 (also known as TAG-1), and other elements have not been identified so far (22, 23).

In children, adolescents, and adults (24), a broad spectrum of autoimmune neurological disease, including paraneoplastic as well as non-paraneoplastic disorders, has been reported to be associated with autoantibodies targeting against VGKC complex, measured by radioimmunoprecipitation or other antibody screening assays (25). While 100 pmol/L is often defined as a threshold for VGKC complex antibody-positive level (24, 26), it is also considered reasonable and significant to make a cutoff for the positive level of antibodies in patients since that high-positive levels (>400 pmol/L) are definitely clinically relevant to AE diseases, while low-positive levels (100–400 pmol/L), which may be less likely to have a probable autoimmune condition, are often believed to be associated with peripheral nerve hyperexcitability syndromes (25, 27). However, this cutoff value should not be strictly viewed as the sole gold standard when it comes to the diagnosis of AE diseases, in that there are also other detection results showing that titers of VGKC complex antibodies were diverse in cohorts with intersubject variability and even fluctuated itself sometimes (23, 28, 29). Studies on limbic encephalitis (LE) found that children patients may have lower levels of VGKC complex antibodies compared to that in adults (30, 31).

#### Leucine-Rich Glioma Inactivated 1

Associating with neuronal and non-neuronal cell membranes (32, 33), LGI1 is a secreted protein, the main known component of the VGKC complex, and is strongly expressed in the hippocampal neuropil where it modulates the VGKC activity (34, 35). In most cases, the autoantibodies associated with VGKC complex are typically directed against LGI1. In a large population-based study, it was found that compared to that of CASPR2, the LGI1 antibody positivity was often correlated with higher VGKC complex antibody values in patients (23).

It has been accepted that mutations in the LGI1 gene took responsibility for autosomal-dominant partial epilepsy with auditory features, which was also termed as autosomal-dominant lateral temporal lobe epilepsy (32, 36, 37). In previous studies, the autoantibodies were thought to interfere with the modulation of LGI1 to VGKC, resulting in the disorder of VGKC activity and thus neuronal hyperexcitability and seizures occurred (21, 35). And then, accumulating AE cases related to LGI1 are reported. LGI1-related LE (also known as LGI1 antibody-associated LE) is the most common non-paraneoplastic LE in adults and is usually thought to be responsive to immunotherapies (21, 28, 38–40). This disease often presents some clinical features like memory deficits, partial seizures, psychiatric disturbance, while insomnia, amnesia, confusion, or faciobrachial dystonic seizures occur occasionally (40–44). Positivity for the LGI1 antibody in cerebrospinal fluid (CSF) rather than serum is thought to be a distinctive indicator of LGI1-LE (40, 45). Discussed in another recent study, LGI1 antibody-associated encephalopathy also showed a rapid clinical improvement after an immune treatment with rituximab, a monoclonal antibody targeting against CD20 (46).

Interestingly, not only are the AE diseases associated with LGI1 found in human but they also occur in animals. In animal model studies, knockout-mice void of LGI1 expression developed lethal epilepsy or neuronal hyperexcitability (47–49), zebrafish with knockdown of LGI1 showed a seizure-like behavior (50), LGI1-mutant rats expressing a missense mutation replicated the spontaneous epileptic syndromes in human (51), and cats with feline temporal lobe epilepsy were thought to be caused by an immune-mediated process, which had been confirmed to be related to LGI1 antibodies (52). The consistency in role of LGI1 antibody in AE diseases between human and animals makes it plausible that LGI1 is involved in the pathogenic process of some AE diseases, in which details are still to be further clarified (33). There comes a general idea that the secreted protein LGI1 can bind to presynaptic VGKC and inhibit inactivation (34). When LGI1 is absent due to the specific gene mutation or decreased by relative antibodies, rapidly closing channels can be caused, and the presynaptic depolarization will be extended, leading to the increased calcium influx. Subsequently, the release of neurotransmitter increases excessively, inducing seizures and AE diseases.

**Figure 1:**
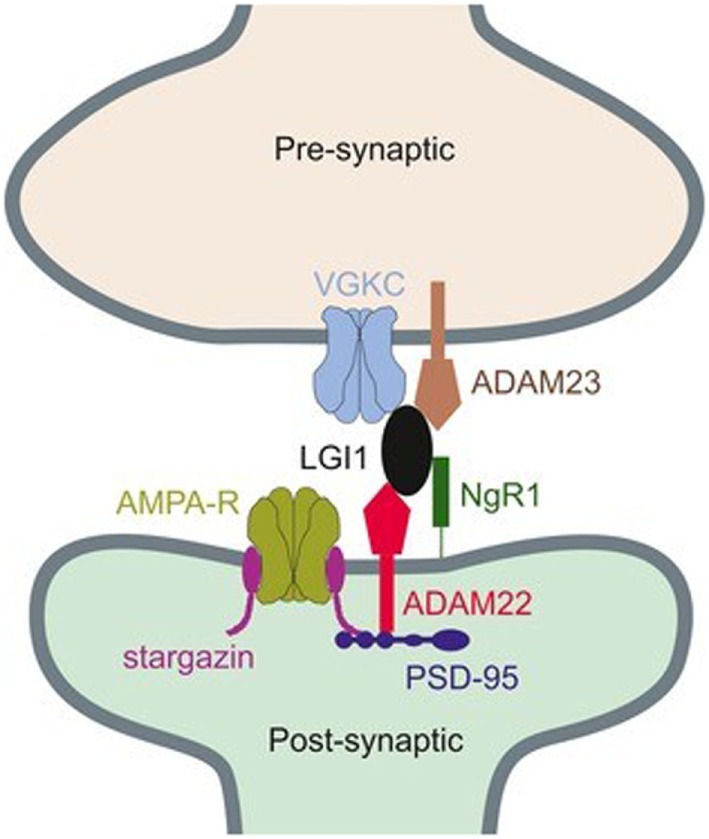
**Connection between presynaptic voltage-gated potassium channel (VGKC) complex and postsynaptic AMPAR through a ternary complex, formed by ADAM22, leucine-rich glioma inactivated 1 (LGI1), and ADAM23 (55)**. Copyright 2017 and Copyright 2015, Wiley’s Open Access Terms and Conditions.

While LGI1 may function in the modulation of the VGKC, it was also found that this extracellularly secreted protein could have an effect on the regulation of 3-hydroxy-5-methyl-4-isoxazolepropionic acid receptor (AMPAR)-mediated synaptic transmission (47, 53, 54). In molecular levels, two epilepsy-related proteins in the brain, ADAM22 and ADAM23, were identified in this study, both of which are members of a disintegrin and metalloproteinase (ADAM) protein family. They were thought to be linked by LGI1 and composed with LGI1 as a ternary complex, which pulled both presynaptic VGKC and postsynaptic AMPAR scaffolds together (Figure 1) (47, 55). The existence of LGI1 makes sure the normal connection between pre- and postsynaptic membrane, stabilizes the synapse, and increases neurotransmission. The absence of ligand–receptor interaction between LGI1 and ADAM22 was found to cause abnormal synaptic transmission and epilepsy in the LGI1 specifically disrupted mice (54). The loss of LGI1 also led to a clear and reversible reduction in the AMPA/*N*-methyl-d-aspartate receptor (NMDAR) ratio, which was further discovered to be a selective decrease in AMPAR synaptic currents (47). It was thought that LGI1 antibodies made exclusive contributions in the pathogenic mechanism of AE diseases, on account of the fact that these antibodies could disrupt the ligand–receptor interaction between LGI1 and ADAM22, by binding the specific epitope of LGI1 which was found to be EPTP repeat domain (54). Allowing for the fact that another domain of LGI1, LRR domain, frequently involves in the protein–protein interaction, it is considered reasonable that LGI1 autoantibodies bind to both LRR and EPTP repeat domains (56). There still exists a question why the inhibition of LGI–AMDAR22 interaction may reversibly reduce the density of synaptic AMPAR. As can be seen in Figure 1, AMPAR and ADAM22 are anchored in the same PSD-95-scaffolding platform but bound to different domains, respectively. When LGI1 and ADAM22 bind together, it is speculated that AMPAR can interact with PSD-95 more stably through its auxiliary subunit transmembrane AMPA receptor regulatory proteins (57, 58). As a result, the loss of LGI–AMDAR22 interaction affects AMPAR transmission, unbalances the regulation of brain excitability and memory storage, and eventually leads to epileptic disorders. Generally, LGI1 may serve as a major determinant of brain excitation and be a key exciting therapeutic target for AE diseases, if the secretion of LGI1 is regulated in a synaptic activity-dependent way (47, 53).

#### Contactin-Associated Protein 2

Found in the last decade, CASPR2 was a transmembrane axonal protein as well as a cell adhesion molecule located at the juxtaparanodes of myelinated axons. It was believed to play a crucial role in the localization and modulation of VGKC for proper nerve impulse conduction and normal axonal excitability regulation (59). Subsequently, immunological investigations in CASPR2 found that the large extracellular sequence consisted of multiple domains, which could be further divided into distinctive subdomains that formed a scaffold to maintain the structure of VGKC (60). Other studies suggested CASPR2 was also involved in the formation of synaptic network as a cell recognition molecule (61). The pivotal role of CASPR2 in epileptic disorders was expanded in 2010 when it was first proved to be a target autoantigen of neural autoimmune disorders (22). Further research never stops. Antibodies against CASPR2 were also found in association with various immune disorders, including encephalitis (62), Morvan’s syndrome (63, 64), peripheral nerve hyperexcitability, and cerebellar ataxia (65). Taken together, these findings demonstrate the multifocal clinical features and complex etiology for underlying processes of autoimmune disorders, and the clinical spectrum of CASPR2 autoimmunity is greatly broadened. Based on the fact that most patients with CASPR2 antibodies are often responsive to clinical immunotherapy, it is highly possible that these antibodies only cause the dysfunction of CASPR2, not neuronal destruction (62). The molecular studies demonstrated that autoantibodies in patients’ CSF were selectively directed against the N-terminal modules of CASPR2, which were thought as the main epitope in multiple subdomains (66). This disruption may neutralize the interaction between CASPR2 and TAG-1. The autoantibodies were preferential to target the axons of inhibitory neurons, which led to a structural alteration of inhibitory postsynaptic scaffold; thereby the perturbation of inhibitory synaptic transmission was induced (61).

#### Other Possible Antigenic Components

Several reports on the epileptic encephalopathy or LE found that the VGKC complex antibodies titers in patients’ serum were elevated, but both LGI1 and CASPR2 antibodies testing were negative (6, 26, 30, 67). In a large serologic study, it was also found that an inescapable proportion of patients were detected with high seropositive VGKC complex antibodies, but these antibodies lacked specificities to LGI1 and CASPR2 (23). While in children presenting with acute-onset explosive seizure disorders or status epilepticus, similar perplexing results are reported (68, 69). Taken together, all of these reports indicate that within the VGKC complex it may still remain some other possible pathogenic elements to be identified, which also play an important part in the modulation of immune system.

### AE Associated with Glutamate Receptor Antibodies

Prevalently located on the membranes of neuronal cells, glutamate receptors are synaptic receptors modulating the synaptic plasticity, a property of the brain thought to be essential for memorizing and learning. Based on the different mechanisms in the way of giving rise to a postsynaptic current, glutamate receptors can be divided into two groups, which are ionotropic glutamate receptors (iGluRs) and metabotropic glutamate receptors. At most times, AE diseases are discussed in association with iGluRs, further divided into NMDAR, AMPAR, and Kainate receptor.

#### *N*-Methyl-d-Aspartate Receptor

*N*-methyl-d-aspartate receptors are glutamate-gated cation channels, with crucial roles in synaptic transmission and neuronal plasticity. Similar to other ion channels, NMDARs are also heterotetrameric complexes, formed by glycine-binding NR1 subunits and glutamate-binding NR2 (NR2A–NR2D) subunits (70), in which NR2A-NMDAR and NR2B-NMDAR subtypes, exclusively located in the postsynaptic membrane compartment (71), are fundamental. Although both NR2A-NMDAR and NR2B-NMDAR subtypes can be detected on the neuronal surface, they share different locations in the synapses. With NR2B-NMDAR mostly extrasynaptic, NR2A-NMDAR is rich in the postsynaptic density of glutamatergic synapses (72). With the development of molecular biology for many years, the role of NMDAR antibodies in AE diseases has been elucidated gradually. It was showed that the pathogenesis of AE diseases with NMDAR antibodies is neither a cytotoxic T cell attack nor complement-mediated neuronal damage (73). In a prospective study, NMDAR antibodies isolated from patients’ CSF were applied to neurons in culture, rapidly leading to the decrease of NR2A-NMDAR and NR2B-NMDAR surface content, and preventing the long-term potentiation of glutamate synapses, while other membrane receptors and channels almost remained unaffected (72, 74). Paving a good way for further studies in pathogenesis of AE diseases and immunotherapy strategies, this study demonstrated that autoantibodies directed against NMDAR would specifically contribute to the lateral displacement of surface NR2A-NMDAR out of synapses and completely block the synaptic plasticity.

The most common AE disease associated with NMDAR is anti-NMDAR encephalitis, which was first described in patients with paraneoplastic encephalitis that may result from ovarian teratoma (75). However, years later, it was shown that anti-NMDAR encephalitis could also be found in the absence of a detected tumor (76, 77), while others gave an explanation that excluding the possibility of detection technical fault, it was the autoantibody produced as an effective immune response that decreased the size to small or even eliminated the tumor completely, leading to a negative diagnosis of tumor. There are still some researchers arguing that the presence of antibodies implies an underlying tumor, on the basis of the phenomenon that tumor is often occult and neurologic disorders typically precede the diagnosis of tumor. The third interpretation also seems plausible that the anti-NMDAR encephalitis may be triggered by other infectious agents, rather than ectopic antigen expression of tumors. Actually, whether the presence of tumor is essential for the anti-NMDAR encephalitis is still fraught with arguments nowadays.

**Figure 2:**
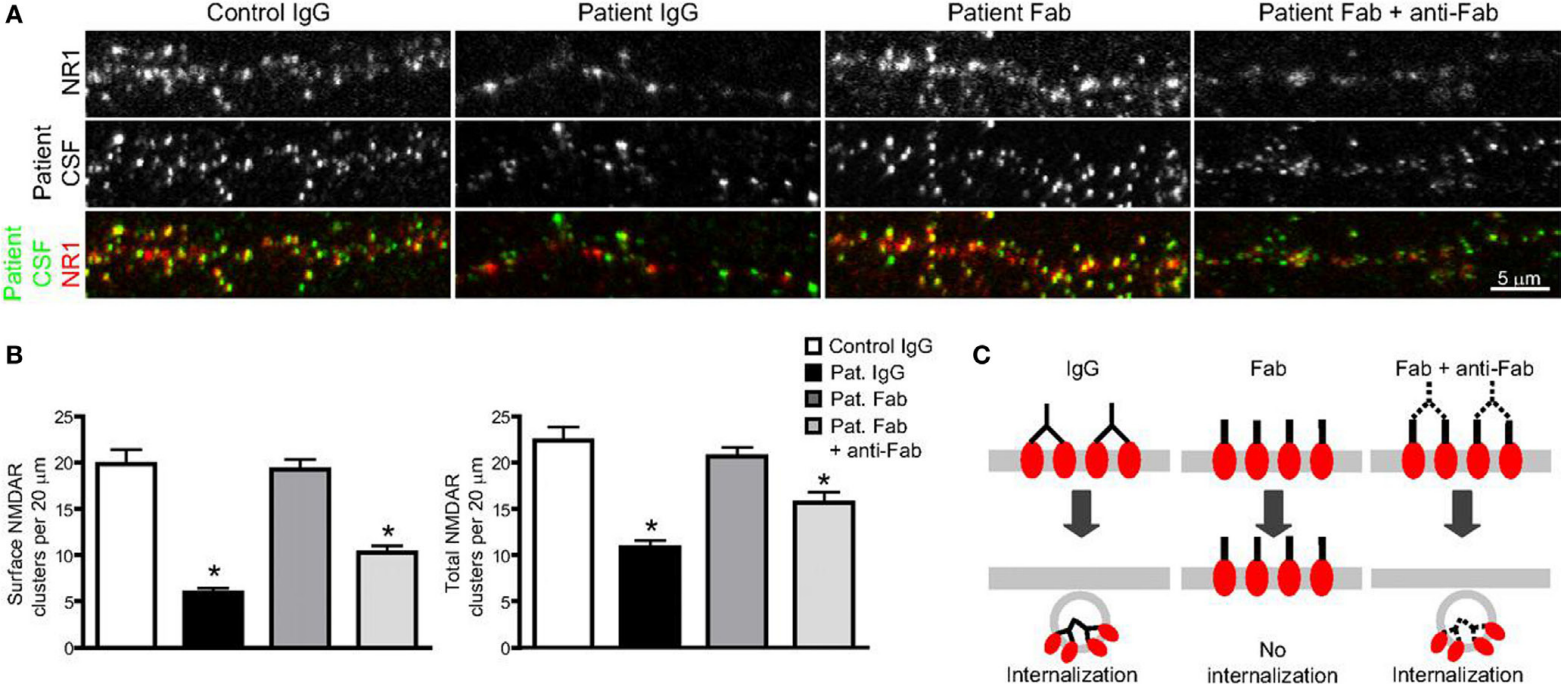
**The binding, crosslinking, and internalization of autoantibodies to *N*-methyl-d-aspartate receptor (NMDAR)**. **(A)** Surface and total NMDAR clusters are immunostained. Treatment with patients’ autoantibodies leads to a decrease of NMDAR cluster both in surface and total (middle left). Treatment with only Fab fragment does not influence the density (middle), while combination with Fab fragments and anti-Fab secondary antibodies together decrease cluster again both in surface and total (middle right). **(B)** Statistical analysis of the effects on surface and total NMDAR cluster density of three different treatments. **(C)** The outline that shows the effect of treatments on surface receptor clusters (74). Copyright permission from the correspondence, Dr. Rita J. Balice-Gordon.

As more and more study results confirm the pathogenicity of NMDAR antibodies, which are proved to be necessary as well as sufficient to cause the loss of surface NMDAR (78–80), the underlying mechanisms are reviewed as follows (Figure 2).

##### Immune Activation (Production of Autoantibodies)

Tumors are presumed to bear the responsibility of autoantibody occurrence at most times. In a review of adaptive autoimmunity in the CNS, it was thought that the tumor was the source of certain unknown self-antigen, which would induce the initial expansion of the T and B cells and contribute to the production of tumor-specific antibodies (82). These antibodies, unfortunately, can cross-react with NMDAR and cause neurological dysfunction. Another trigger for the initial occurrence of autoantibodies may be pathogenic infection, in the non-paraneoplastic form. The infection was considered to activate the immune response in the way of molecular mimicry that infectious microbes or virus expressed antigens sharing structural homology with self-antigens and thus initiated the immune response against antigens both in microbes and human bodies (83, 84). A population-based case analysis found that within 2 weeks before hospital admission, two-thirds of patients had had a viral-like illness, which was thought to be a trigger (77). Additionally, several reports found a progression to encephalitis in patients, with the diagnosis of herpes simplex virus followed by the production of NMDAR antibodies (85–87). Moreover, in the 1-year follow-up study, it was suggested that prodromal Epstein–Barr virus reactivation and following cascade may also boost the immune response and facilitate the production NMDAR antibodies (88).

##### Blood–Brain Barrier (BBB) Disruption

Normal brain is shielded by the BBB. Animal models showed that sensitized T cells could cause greater permeability of BBB, although hardly did we know about their exact phenotype and whether they are actually involved in this BBB disruption (89). The BBB disruption permits the infiltration of autoreactive memory B cells, plasma cells, and other relative immunological cells. Also, in this process, specific NMDAR antibodies secreted by plasma cells can move across the BBB, and then together with those secreted by passed plasma cells in CSF, bind to self-antigen and disable the normal function of NADAR. BBB is so important in the immune system that it effectively protects the brain from most pathogens. Extensive literature on BBB dysfunction in epilepsy is reported, mostly emphasizing the causes such as inflammation, channel dysfunction, or ionic disturbances, all of which may trigger an innate immune response to disrupt BBB integrity (2, 90, 91). A multicenter, population-based prospective study suggested that the NMDAR autoantibodies, almost exclusively found in the serum or CSF of individuals with AE, were also present in the serum of health controls, of which 10% showed high seroprevalence (92). Here comes the question why positive NMDAR antibodies do not show clinical features in these healthy individuals. Does it mean NMDAR antibodies are insufficient to initiate AE diseases? Actually, this may be attributable to the BBB integrity that prevents the ingress of pathogenic antibodies in healthy people, as is shown in the correlation between clinical significance and perturbations of BBB function (92). However, whether NMDAR antibodies could lead to a developmentally leaky BBB is still unclear.

##### Attenuation of NMDAR Function

**Figure 3:**
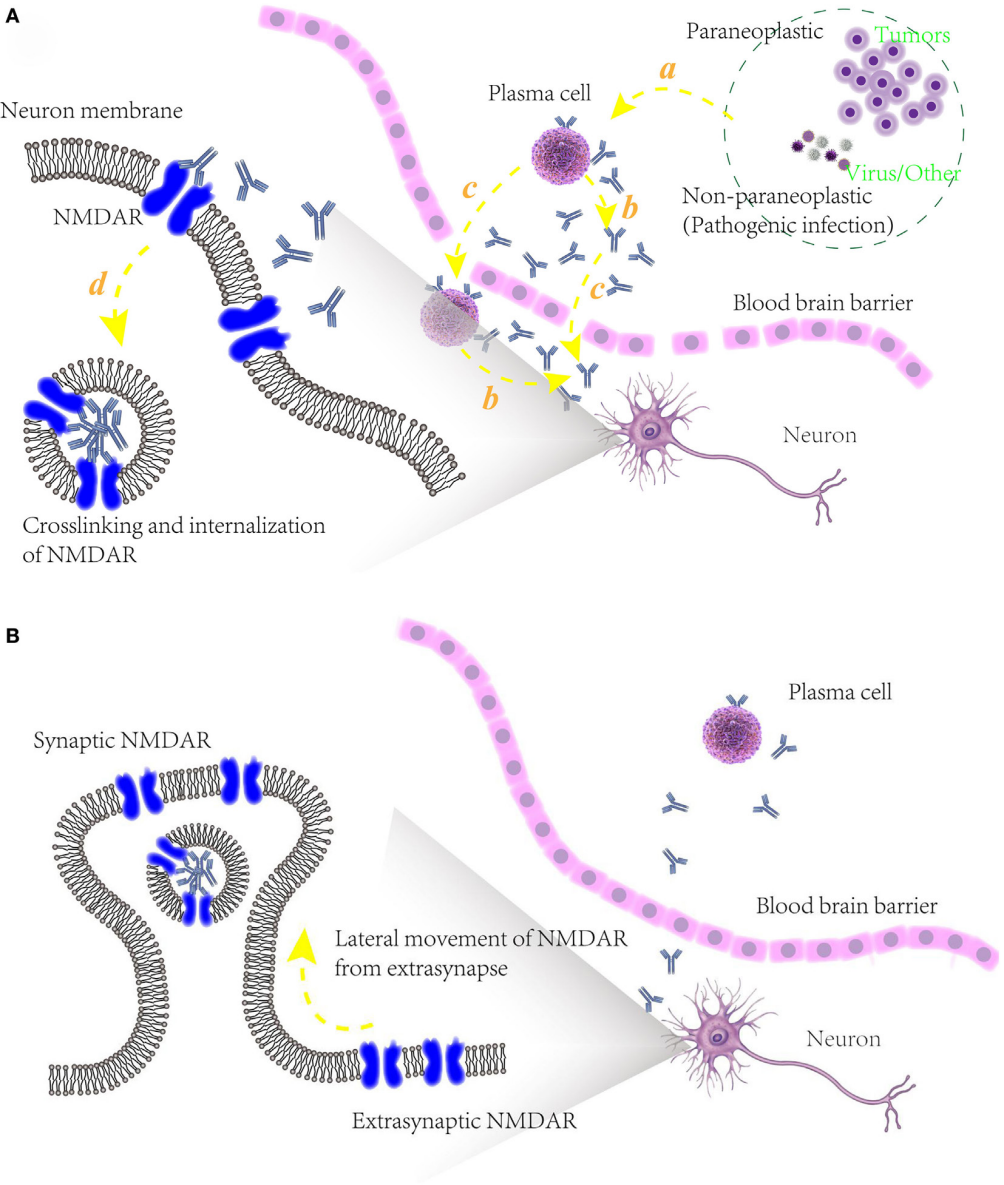
**(A)** Schematic models of potential pathogenic mechanism of autoimmune epilepsy (AE) diseases associated with *N*-methyl-d-aspartate receptor (NMDAR) autoantibodies. (a) Initiation of B cells, triggered by paraneoplastic agents like tumors or non-paraplastic factors like virus or infection. (b) Secretion of specific antibodies to NMDAR. (c) Blood–brain barrier disruption, allowing the infiltration into cerebrospinal fluid of plasma cells, specific antibodies and maybe other relative immunological cells. (d) Attenuation of NMDAR function, caused by the binding of NMDAR with antibodies and internalization of presynaptic NMDAR; **(B)** underlying mechanism of recovery of some AE diseases associated with NMDAR autoantibodies. After an elimination of NMDAR densities in synapse, unblocked and functional NMDARs in extrasynapse move laterally into preexisting synapses in a compensatory way, maintaining the stability of neuronal network activity.

When NMDAR autoantibodies move across the transiently compromised BBB to CSF or are secreted by passed plasma B cells in the CSF, the binding to NMDAR will cause a selective and reversible decrease in NMDAR surface protein and cluster density, which attenuated the NMDAR function and resulted in neural hypoactivity (74, 93). In a molecular mechanism study, it was proposed that the induction of a rapid dispersal of NR2A-NMDAR by NMDAR antibodies inhibited the downstream interaction between extracellular domains of NR2A subunits and prevented dynamic retention (94). The mechanism of this decrease and attenuation is thought to be either direct pharmacological blockade of the NMDAR or antibody-mediated internalization and downregulation of surface NMDAR, in a time-dependent and activity-independent manner (95). However, the reversibility of most AE diseases associated with NMDAR, irrespective of the duration of symptoms, suggests the indispensable role of internalization in this process rather than completely irreversible blockade and destruction (77, 78). To identify the internalization of surface NMDAR, the Fc IgG domain was enzymatically removed to generate Fab fragments *in vivo* studies (74, 78). It was found that these monovalent Fab fragments bound specifically to NR1 clusters, but the density of NMDAR cluster did not decrease compared with that in neurons treated with control IgG (Figures 3A,B), which meant no receptor internalization. In contrast, when these Fab fragments were combined with anti-Fab secondary antibodies together, forming a similar conformation to unmodified antibodies, NMDAR cluster density and surface protein in neurons lowered significantly (Figures 2A,B). This study made a detailed explanation that in the internalization process of NMDAR, autoantibodies bound, capped, and cross-linked with receptors, and then led to the loss of NMDAR (Figure 2C) (74). The elimination of NMDAR-mediated synaptic function suppressed the induction of long-term potentiation and eventually resulted in episodic memory impairment (96), which was a typical feature of AE.

##### Recovery

For a long time, it has been thought that NMDAR is always in a static state and tightly anchored in a highly organized and stable synaptic membrane surface. Further studies, however, challenge this view. Early in 1997, a molecular level study adopted NMDAR antagonist 2-amino-5-phosphonovaler-ate to block receptors, caused a 380% increase in the number of NMDAR clusters at synaptic sites, and also led to a dramatic shift in the pattern of NR1 immunoreactivity, which was later proved to be indeed a shift in the distribution rather than number change due to the fact that the generalized amount of NR1 at all sites was almost same (97). To examine the mobility of NMDAR at hippocampal synapses, open-channel blocker (+)-MK-801 was used to irreversibly block NMDAR in two distinct ways (81). By coapplication of NMDA and MK-801, all NMDARs in synapse and extrasynapse were completely and irreversibly blocked, and NMDAR-mediated excitatory postsynaptic current (EPSC) showed no recovery even 30 min after MK-801 removal. While another way, applying MK-801 during synaptic stimulation was used to selectively block synaptic receptors, and in this case, there was a consistent and significant recovery in EPSC after the following MK-801 removal. The recovery, which could not be attributed to the new synapse formation, neither the insertion of new formed receptors into the membrane nor recruitment of receptors into existing synapses, was thought to be consistent with the lateral movement of unblocked and functional NMDAR from extrasynapses into preexisting synapses (81). It implied that extrasynaptic and synaptic membranes may share relatively equal NMDAR densities, and when NMDAR function was attenuated, extrasynaptic NMDAR serving as receptors in storage would move laterally into synapses, restore the normal physiology function of synapse in a compensatory way.

Similar to the NMDAR antagonist, NMDAR autoantibodies may also have a positive effect on the increase of NMDAR clusters at synaptic site. However, this phenomenon would be undetectable on account of the fact that the surface NMDARs are decreased greatly by the antibody-mediated internalization and this decrease is over the natural range of homeostatic plasticity of synapses density, which maintains the stability of neuronal network activity (98).

#### AMPAR

AMPAR are assemblies of four core subunits designated as GluR1–4 and mediate most excitatory fast synaptic transmission in the CNS (99). In a study, it was found that antibodies directed at one or both of GluR1 and GluR2 subunits of AMPAR were associated with LE (100). Also, antibodies directed specifically against GluR3 subunit were found in patients with different types of epilepsy (101). It was thought that AMPAR antibodies bind to an extracellular region on the receptor (100), which was further defined to be the bottom lobe of an amino-terminal domain, an extracellular part of AMPAR (102).

Similar to that in AE with anti-NMDAR antibodies, the pathogenesis of AE with anti-AMPAR antibodies was proposed that in the way of increasing internalization and degradation of surface AMPAR clusters, anti-GluR1 or anti-GluR2 antibodies in patients selectively eliminated the surface amount and synaptic localization of AMPAR (100). This perturbation resulted in the decrease of homeostatic plasticity in inhibitory synaptic transmission and thereby the intrinsic excitability increased, which led to the occurrence of AE diseases (47, 103). Furthermore, it was also found that in normal neurons AMPAR are constantly cycling between the cell membrane and intracellular compartments (104). When antibodies were present in neurons, the balance of internalization and reinsertion would be disrupted, contributing to the accumulation of internalized AMPAR that may be further targeted to early or recycling endosomes, or transferred to lysosome for degradation (103).

### AE Associated with Gamma-Aminobutyric Acid (GABA) Receptor Antibodies

Gamma-aminobutyric acid receptors are the main inhibitory receptors throughout the mature vertebrate CNS and can be divided into two classes further: GABAA and GABAB receptors.

#### GABAA Receptors

GABAA receptors are ligand-gated ion channels, predominantly mediating most of the fast inhibitory neurotransmission in the brain, and are pharmacological targets of many clinically important drugs (105). Antibodies to the GABAA receptors have been reported in a number of cases, including some AE diseases like severe encephalitis with seizures, refractory status epilepticus, and epilepsia partialis continua (106–108). The antibodies cause a selective reduction of synaptic GABAA receptors, possibly through the crosslinking and internalization of antibody–receptor complexes (109), in the similar way as described for NMDAR and AMPAR.

#### GABAB Receptors

Acting as functional heterodimers composed of two subunits GABAB1 and GABAB2, G-protein coupled GABAB receptors are metabotropic transmembrane receptors, and mediate pre- and postsynaptic inhibition. A previous study showed that anti-GABAB receptors antibodies are predominantly associated with LE and seizures (110), most times in the presence of diagnosed small cell lung cancer (111, 112). Unlike the underlying pathogenic mechanism of AE diseases with anti-NMDAR or anti-AMPAR antibodies, it was recently suggested that GABAB receptors antibodies directly blocked the receptors without internalization (113). However, considering the perplexity of the pathogenesis of AE associated with GABAB receptors, in which both immune system and humoral factors are involved, it is too ambiguous to draw a conclusion about whether immune elements or humoral factors are the exact primary mechanism. In another study, it was suggested that CD8+ T cells may play a pathogenic role and two possible processes were proposed (114). The first one is that autoimmune reaction mediated by primary CD8+ T cells against some certain cells such as mesial temporal lobe neurons or other parenchymal cells may trigger humoral autoimmunity toward GABAB receptors. While another process, which is considered by author to be more creditable, is that expression of GABAB receptors by some abnormal cells initiates the pathogenic immune response. Once these generating autoantibodies bind to GABAB receptors specifically, pre- and postsynaptic GABAergic inhibition would be disturbed, and the modulation of physiology would be changed, thereby inducing some pathologic behaviors, which are commonly seen in AE diseases (115).

## AE Studies with Antibodies to Intracellular Antigens

Early since 1980s, in patients with immune-associated disorders, autoantibodies directed to specific intracellular antigens have been discovered. In recent years, studies on new antibodies against intracellular antigens were further developed. In these intracellular antigens, what were observed and studied most frequently are proteins such as Hu, Ri, Yo, and glutamic acid decarboxylase (116–119), while other antigens like CV2, Ma2, amphiphysin, or Tr are also identified (120–123) (Table 1). Generally, autoantibodies targeting against intracellular antigens are almost exclusively found in paraneoplastic CNS disorders (124, 125). It was prevalently accepted before that the expression of these antibodies was a part of an effective immune response initiated to control tumor growth but misdirected to bind against intracellular antigens, which were identically shared by tumor cells and neural tissues, and thus vigorous neurological dysfunction occurred (126, 127). While further immunohistochemical studies demonstrated that these paraneoplastic disorders associated with intracellular antigen were mediated by cytotoxic T cells rather than by the antibodies themselves (73, 128, 129). Indeed, this conclusion does not deny the involvement of antibodies in this process but suggests a scenario, as is mentioned, that the production of autoantibodies is an epiphenomenon triggered by T cells and plays a secondary role in the pathomechanism. However, this point of view was partly challenged by the findings of a recent anti-Yo antibody study, which claimed the primary role of anti-Yo antibody in disease pathogenesis (130). When incubating CSF containing anti-Yo antibodies with rat cerebellar slice cultures *in vitro*, researchers found that Purkinje cell death was triggered and it was also demonstrated that the interaction between antibody and Yo antigen directly resulted in the death of Purkinje cell, without the existence of any immune cells including activated T cells (130, 131). Nevertheless, the respective roles and interrelationships of antibodies and T lymphocytes in diseases pathogenesis have remained uncertain so far.

**Table 1:** **Intracellular antigens found in autoimmune epilepsy (AE) diseases**.

Antigens	Functions *in vivo*	Associated tumors and other disorders
Hu	RNA-binding protein, crucial in the development and maintenance of neuronal phenotype	SCLC, ovarian tumor, neuroblastoma, etc. (134–136) Limbic encephalitis (LE), PCD, paraneoplastic encephalomyelitis, etc. (118, 137–139)
Ri (Nova)	Highly conserved and neuron-specific RNA-binding nuclear protein, possibly involving in the developmental biology of the motor system (140)	Breast and gynecologic cancers Opsoclonus myoclonus syndrome, brainstem encephalitis, etc. (141, 142)
Yo	May serve as an essential role in Purkinje cell survival^a^	Ovarian cancer, breast cancer (130, 143) POMA, PCD (117, 144)
Glutamic acid decarboxylase	Key enzyme in the synthesis of inhibitory neurotransmitter gamma-aminobutyric acid (GABA), modulating the function of GABAergic neurons	Thymoma SPS, cerebellar ataxia, LE (107, 116, 145–148)
CRMP5	Important protein in the axon formation	SCLC, thymoma Encephalomyelitis, cerebellar ataxia (120, 149, 150)
Ma2	Protein encoded by the *PNMA2* gene^a^	Testicular cancer, breast cancer LE, brainstem encephalitis (121, 151, 152)
Amphiphysin	Vesicle-associated protein on synaptic terminal, regulating the recruitment of dynamin to sites of endocytosis	Breast cancer, SCLC (122, 153) SPS, PCD, encephalopathy encephalomyelitis, LE, etc. (154–156)
DNER	Single-pass type I transmembrane protein, mediating PC–Bergmann glial interaction during cerebellar development	Hodgkin’s disease PCD (123, 157)

*^a^Function of the intracellular antigens has not been elucidated by in vivo analysis*.

*SCLC, small cell lung cancer; PCD, paraneoplastic cerebellar degeneration; POMA, paraneoplastic opsoclonus-myoclonus ataxia; CRMP5, collapsin response mediator protein 5, the target antigen of anti-CRMP5 (anti-CV2); DNER, delta notch-like epidermal growth factor-related receptor, the target antigen of anti-Tr; SPS, stiff-person syndrome*.

In most cases, the presence of these autoantibodies can be a useful diagnostic indicator for underlying malignancy since there exists a good correlation between them (158, 159). Compared to that with antibodies against surface antigens, AE diseases with antibodies directed toward intracellular antigens are often considered to be poorly responsive to immunologic therapies (132, 160, 161). This is probably related to the pathologic features that they are often subacute and associated with neuronal loss mediated by T cells (125, 126).

## Immunotherapy in Human AE Diseases

### Diagnostic Assessment

In a refinement of case reports for the identification of diseases associated with antibodies, Tables 1 and 2 are sorted out for the reference of the clinical diagnosis. However, as has been mentioned, current clinical methods in diagnosis are still limited, considering the unknown self-antigen or the undiscovered features in various types of AE diseases. In this case, an all-round diagnosis and detection on all known autoantibodies may be an effective but not cost-efficient choice. Other autoantibody testing technology and other progress in molecular mechanisms of AE are needed for a more precise and timely diagnosis.

**Table 2:** **Autoantibodies in human epilepsy and animal models**.

Specific antibody		Human epilepsies in which the specific antibody is found	*In vitro*	*In vivo* induction of epilepsy
Antibodies to proteins associated with voltage-gated potassium channels	Anti-leucine-rich glioma inactivated 1 (LGI1) Anti-contactin-associated protein 2 Anti-contactin-2	Limbic encephalitis (24, 27, 40, 132) Epileptic encephalopathy (12, 26, 46) Focal epilepsy (133) Human autosomal-dominant lateral temporal lobe epilepsy (32, 36, 37)	Acute neuronal cell death and loss (may be caused by complementary-mediated mechanisms) (29)	Yes, in rats with heterozygous LGI1 mutation (51)
Antibodies to ion channels and receptors	Anti-NMDA receptor	Anti-*N*-methyl-d-aspartate receptor encephalitis (80, 88)	A selective and reversible internalization of surface receptors in cultured neurons (80)	Yes, in mice infused with continuous patients’ cerebrospinal fluid (80)
	Anti-AMPA receptor	Anti-AMPAR encephalitis (102, 103)	Degradation of surface AMPAR clusters (47, 103)	\
	Anti-GABAA receptors	Encephalitis (106–108)	Selective reduction of synaptic GABAA receptors (may also be in the way of crosslinking and internalization) (109)	\

### Treatment

**Figure 4:**
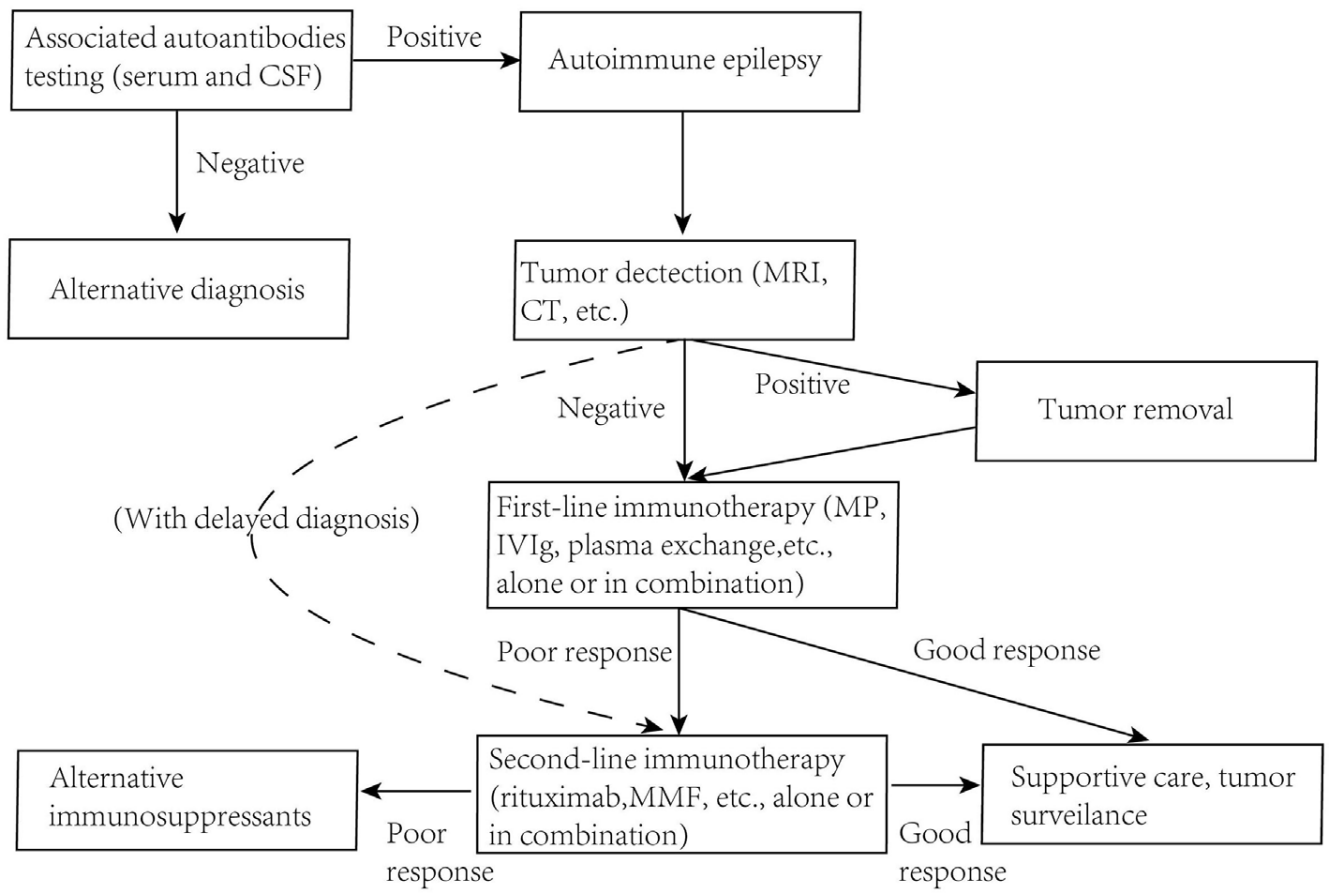
**Guidelines for immunotherapy of autoimmune epilepsy diseases**. MRI, magnetic resonance imaging; MP, methylprednisolone; MMF, mycophenolate mofetil.

While clear pathogenic mechanisms of AE diseases will possibly help us adopt good clinical immunotherapies, conversely it may also be true that a good result of clinical immunotherapy trails, which could imply the potential mode of action, will help us to get a better understanding of the exact molecular mechanism of antibody pathogenicity. Despite there are no strictly controlled clinical trials in AE diseases, plentiful case reports of common entities with good clinical immune-therapeutic outcomes could imply the efficacy of certain treatments in a common way. Although the precise protocols of immunotherapy for different AE diseases vary in different cohorts with different ages or even genders, a brief but generic guideline could be extracted based on these clinical treatments (Figure 4).

There is a general consensus that early diagnosis and immunotherapy are needed since they may actually limit the duration or severity of the illness and often promise a better outcome (12–15). Pulsed intravenous steroids, followed by human intravenous immunoglobulins (hIVIg), corticosteroids, or plasma exchange is taken as a first-line immunotherapy. In patients with an underlying tumor, these treatments may enhance effectiveness and leave fewer neurological relapses if the tumor was firstly removed (77, 83, 162). There are also second-line immunotherapy options, like rituximab, cyclophosphamide, azathioprine, methotrexate, and mycophenolate mofetil (MMF), applied to patients without a tumor or with delayed diagnosis (40, 163–165). Plasma exchange and hIVIg as first-line immunotherapies are believed to clear pathogenic antibodies rapidly and halt the ongoing damage to human CNS (38). Two AE diseases with relative high frequency are discussed here, which may also be disorders best studied. The therapeutic approach is usually done following the strategies below.

#### Voltage-Gated Potassium Channel-Limbic Encephalitis

At present, the therapy widely accepted and adopted for severe epilepsy in LE with antibodies to VGKC complex is steroid therapy regimen, which could benefit in faster decline in antibody titers and improvement in cognitive function (17). However, the potential risk of some severe adverse effects such as acute liver failure may often limit the clinical use (6). Immunotherapy can be of utility in patients with VGKC-LE of all ages, although it is possible that long-term therapy is better than those with short period.

The common immunotherapies applied were hIVIg, plasma exchange, intravenous methylprednisolone (MP) pulses, and immunosuppressive treatments with rituximab, prednisolone mycophenolate, or MMF, alone or in various combinations. In a small clinical group of LE-VGKC patients, most patients (13/18, 72.2%) became seizure-free with monthly intravenous MP pulses, while hippocampal atrophy and poor memory had developed in some individuals, implying a need for more intense immunological treatment (6).

In contrast, in an open-label prospective study, long-term immunotherapy with a combination of plasma exchange (50 ml/kg), IVIG (2 g/kg), and intravenous MP (1 g ×3), followed by maintenance oral prednisolone (1 mg/kg) seems more promising (38). All nine patients showed clinical improvement and sustained immunological remission with this immunotherapy regimen, and timely diagnosis seemed to ameliorate hippocampal atrophy-associated cell death and clinical disability, while one patient developed septicemia later and another thrombosis due to adverse events of plasma exchange (38). Controlled clinical trials were performed by the comparison in immunotherapy results between treatment with steroids alone and combination of steroids and hIVIg, and it was found that patients with combination therapy were more likely to have a reduced relapse rate (165). In this case, two useful approaches were proposed when treating patients with first-line immunotherapy: the first one, treating with one first-line agent, and when a patient relapses or shows insufficient response, adding another agent; the other approach, treating with combination therapy in the beginning (165).

#### Anti-NMDAR Encephalitis

Although the effectiveness of immunotherapy and its long-term effect are still to be established, available evidence indicates that second-line treatment is often adopted and has been found effective when first-line immunotherapy fails. To analyze systematically the effects of sequential immunotherapy (first-line, second-line), an observational cohort study with at least 4 months follow-up was done. Four hundred seventy-two of 501 patients (94.2%) with NMDAR encephalitis received first-line immunotherapy and tumor removal when applicable, and then 251 (53.2%) had substantial neurological improvement while 221 (46.8%) had little or no response. Of these patients who did not respond to first-line immunotherapy, second-line immunotherapy with rituximab, cyclophosphamide, or both were taken to some patients, and 84 of 125 patients (67.2%) had a good outcome and a decreased occurrence of relapse (166). Generally, once the diagnosis of anti-NMDAR encephalitis is confirmed, immunotherapy including high-dose intravenous corticosteroids, IVIg, plasma exchange, azathioprine, cyclophosphamide, MMF, tacrolimus, methotrexate, and monoclonal antibodies like rituximab and others should be used in sequence or in combination (167).

## Future Directions

Although the results of clinical immunotherapy are promising so far, the trails are often complicated and vary widely in methodology. Furthermore, the immune treatments discussed here, including first-line and second-line options, are almost either immunosuppression or immunomodulation, which remain to be the standard of care clinically, and further rationale with improved efficacy is to be continued. Based on the known mechanisms of AE and other similar autoimmune diseases, however, there also exist a number of other potential therapy targets that are promising for the coming of novel immune therapeutics and can be complementary strategies to the current symptomatic treatments.

### Inducing Antigen-Specific Tolerance

Although the technology of antigen-specific tolerance induction has been proposed and applied widely in many autoimmune diseases for functional modulation of lymph cells (168), it has been seldom or never reported in the therapeutic trials of AE diseases, probably for the reason that the innate self-antigen varies in different AE. Finding proper tolerance inductions may be another difficulty, which should be secure as well as effective at the same time. However, this direction is still worth trying since the clinical efficacy of existing immunotherapy to AE diseases has a distance to the ideal therapeutic expectation. As an example, taking advantage of the trait that repeated sequences from parasite are of essence to evade the host immune system, researchers linked repetitive structures from specific parasite proteins to defined self/T-cell epitopes together, tested and verified successfully the effectiveness to induce antigen-specific tolerance (169).

### Targeting B Cell Surface-Specific Molecules Selectively

Targeting cell surface molecules that are not specific for most B-lineages cells, take rituximab, for example, would exert a broad adverse influence on immune cell population, and thus human body is more likely to be invaded and attacked by external infections. While in this case, selectively targeting immune cells that secret pathogenic autoantibodies and simultaneously preserving immune-protective cell population is actually the most ideal strategy (170). According to a preclinical study, some certain molecules are especially highly expressed or are only present during defined B-cell differentiation stages, which could be the underlying targets of antigen-specific immunotherapy (171). For example, CD269 (also known as B-cell maturation antigen) is a cell surface receptor preferentially expressed in mature B lymphocytes, while CD319 (SLAMF7) is another preferential protein of plasma cells and is often used as plasma cell marker nowadays (172, 173). Humanized antibodies specifically targeting against CD269 have been invented as a medicament in the treatment of disorders associated with the presence of pathogenic B cells (174). However, so far neither these two B cell preferential surface molecules have been found feasible in immunotherapy of AE. There may be still a long way to go before this ideal treatment comes true.

### Increasing the Capacity of Immune-Regulation

Given the complex interplay of innate and adaptive immunity in autoimmune diseases, reconstituting the immune system may come before the blocking of immune activation during the course of developing AE (170). As mentioned above in NMDAR-associated AE diseases, when NMDAR antagonist selectively taken at synaptic site, compensatory NMDAR receptors upregulation would get from extrasynapses if the antagonist was removed next (Figure 3D). In this regard, it is well demonstrated that the ability of homeostatic plasticity of synapses density in our body is potentially strong, which also implies the possibility of immune system reconstruction.

### Resolving the Brain Inflammation and Repairing BBB

Despite whether autoantibodies could lead to a developmentally leaky BBB is still unclear, what we could be sure is that mounting evidence points to a critical role of BBB dysfunction in immune pathogenesis of AE diseases, so blocking excessive inflammatory processes in the brain and restoring the function of BBB may also be a right choice to increase the threshold of AE occurrence. Multiple functional small molecules have already been proposed and sorted out in a perspective review, which are designed to resolve the brain inflammation and thus repair the disrupted BBB (175), and they were not required strictly to penetrate into the brain since the main target is systemic-BBB inflammation rather than neurons inside (90). Furthermore, artificial BBB (176) could also be an efficient substitute for the damaged BBB to reduce the likelihood of recurrent epilepsy even with the presence of autoantibodies out of the brain.

## Discussion

At present, any discussion of the exact definition, relevant pathogenic mechanisms, and specific immunotherapies of AE diseases is fraught with difficulties. Similar to that of many common CNS diseases, the exact pathogenesis of AE is hard to be ascertained since it can be caused by lots of pathogenic processes. Generally speaking, knowledge of potential pathogenic mechanisms in AE diseases could provide valuable insight into the process of developing disease, allow more precise and valuable diagnosis, and enable development of clinical immunotherapy.

However, there is still a long way to go. Whether AE diseases are actually caused by immune system is debatable nowadays, although most researchers hold positive attitudes toward the role of immune system in AE diseases. Therefore, before the pathogenic mechanism of developing AE diseases is elucidated elaborately, it is still rash to arrive at a conclusion on the pathogenic origin of AE. Even in some well-studied AE diseases, it is still not fully understood about the exact role of autoantibodies and relative contribution of B and T cells.

Another difficulty we are facing now is that the mechanisms of most immunotherapies functioning in AE are not clear now, thus potential adverse effects and hidden danger are posed to patients, where individual discrepancies vary. It was well suggested that the clinical therapy of hIVIg is generally of preferable tolerance in patients, with common adverse effects like headache, fever, vomiting, or local skin problems at the site of cannula insertion in 25.5% of the total cohort (177). While for the choice of plasma exchange, unique challenges in patients should be taken into consideration such as patients’ poor cooperativity or autonomic instability, and other complications, especially in the crowd of children. Furthermore, most immunotherapies discussed are recommended based on most clinical trials while the optimum dosing, frequency, and duration of immunotherapy for each kind of AE disease are still to be further studied.

Despite all these limits, it is still very exciting to witness the great leaps we have done in the study of AE diseases over the past decades. No matter which stage of disease research we are in, it is never late to go further for the elucidation of precise pathogenesis and specific immunotherapy, in consideration of the patients who are now suffering from AE. As we keep going, learn more about the molecular pathogenesis, the ultimate goal of early, specific immunotherapy in selected patients to prevent progressive and permanent brain damage will become a few steps closer.

## Conclusion

As outlined, AE is an emerging disease with characteristic of underlying immune pathogenesis and featured by seizures and cognitive impairment clinically. The accumulating discoveries of new self-antigens have greatly broadened the clinical spectrum of AE and promoted further development of studies on pathogenesis of diseases. Clinicians should be alert to any clinical evidence of an autoimmune cause, even in the absence of known autoantibodies discussed here. More effective immunotherapies are needed to facilitate the recovery of AE diseases.

## Author Contributions

ZF, YY, XC, WZ, YX, YC, ZL, and WY participated in the manuscript design, searched databases, extracted and assessed studies, and helped to draft the manuscript. ZF wrote the manuscript. WY conceived the initial idea and the conceptualization, participated in the data extraction and analysis, and revised the manuscript. YC and ZL also participated in revising the manuscript. All authors read and approved the final manuscript.

## Conflict of Interest Statement

The authors declare that the research was conducted in the absence of any commercial or financial relationships that could be construed as a potential conflict of interest.
